# Cardiac Magnetic Resonance Imaging Findings in Patients With Chronic Kidney Disease and End-Stage Kidney Disease: A Systematic Review and Meta-Analysis

**DOI:** 10.7759/cureus.51672

**Published:** 2024-01-04

**Authors:** Deepak Chandramohan, Rhoshini Rajasekaran, Raghunandan Konda, Ashwini Pujari, Sreekant Avula, Megan Bell, Sujith K Palleti, Apoorv Deotare, Roopa Naik, Atul Bali, Prathap Simhadri, Harkesh Arora, Nihar Jena

**Affiliations:** 1 Nephrology, University of Alabama at Birmingham, Birmingham, USA; 2 General Medicine, PSG Institute of Medical Sciences and Research, Coimbatore, IND; 3 Diabetes, Endocrinology, and Metabolism, University of Minnesota, Minneapolis, USA; 4 Libraries, University of Alabama at Birmingham, Birmingham, USA; 5 Nephrology, Louisiana State University Health Sciences Center, Shreveport, USA; 6 Nephrology, Montgomery Kidney Specialists, Montgomery, USA; 7 Medicine, Geisinger Commonwealth School of Medicine, Scranton, USA; 8 Internal Medicine/Hospital Medicine, Geisinger Health System, Wilkes-Barre, USA; 9 Internal Medicine/Nephrology, Geisinger Medical Center, Danville, USA; 10 Internal Medicine/Nephrology, Geisinger Health System, Wilkes-Barre, USA; 11 Internal Medicine/Nephrology, AdventHealth, Florida State University College of Medicine, Daytona Beach, USA; 12 Hospital Medicine, Lovelace Medical Center, Albuquerque, USA; 13 Cardiovascular Medicine, Wayne State University, Pontiac, USA

**Keywords:** end-stage kidney disease, t1 mapping, myocardial fibrosis, cardiovascular magnetic resonance, chronic kidney disease

## Abstract

In this systematic review and meta-analysis, we explored the utilization of cardiac magnetic resonance imaging (CMR) to detect fibrotic changes secondary to uremic cardiomyopathy during the early stages of chronic kidney disease (CKD) and in patients with end-stage kidney disease (ESKD). Uremic myocardial fibrosis can lead to arrhythmia and heart failure, and it is important to detect these changes. CMR offers a noninvasive way to characterize the severity of cardiac remodeling. A comprehensive search of multiple electronic databases was conducted. Studies were divided according to scanner field strength (1.5 or 3 Tesla). The random effects model was used to calculate the pooled mean, 95% confidence interval (CI), standard error, and standardized mean difference (SMD). The I2 statistic was used to assess the heterogeneity between study-specific estimates. The search retrieved 779 studies. From these, 20 studies met the inclusion criteria and had 642 CKD patients (mean age of 56.8 years; 65.2% males; mean estimated glomerular filtration rate (eGFR) of 33 mL/min/1.73 m2) and 658 ESKD patients on dialysis (mean age of 55.6 years; 63.3% males; mean dialysis duration of 3.47 years). CKD patients had an increased left ventricular mass index (LVMi) compared to controls, with an SMD of 0.37 (95% CI: 0.20-0.54; I2 0%; p-value <0.05). ESKD patients also had increased LVMi compared to controls, SMD 0.88 (95% CI: 0.35-1.41; I2 79.1%; p-value 0.001). Myocardial fibrosis assessment using T1 mapping showed elevated values; the SMD of native septal T1 values between CKD and controls was 1.099 (95% CI: 0.73-1.46; I2 33.6%; p-value <0.05), and the SMD of native septal T1 values between ESKD patients and controls was 1.12 (95% CI: 0.85-1.38; I2 33.69%; p-value <0.05). In conclusion, patients with CKD and ESKD with preserved left ventricular ejection fraction (LVEF) have higher LVMi and T1 values, indicating increased mass and fibrosis. T1 mapping can be used for the early detection of cardiomyopathy and as a risk stratification tool. Large, randomized trials are needed to confirm these findings and determine the effect of long-term dialysis on cardiac fibrosis.

## Introduction and background

Cardiac disease is a significant cause of mortality in almost 50% of patients with chronic kidney disease (CKD) and end-stage kidney disease (ESKD). Cardiovascular disease risk occurs independently of comorbidities such as hypertension and diabetes [1]. The term uremic cardiomyopathy is commonly used to describe changes to the cardiac structure that occur in kidney disease, and it involves two distinct processes: left ventricular hypertrophy and myocardial fibrosis. Several factors related to preload, afterload, uremic toxins, interleukin 1 alpha (IL-1α), interleukin 6 (IL-6), carnitine deficiency, parathyroid hormone imbalance, high FGF-23, reduced serum Klotho, high circulating phosphate, volume overload, anemia, increased hepcidin, erythropoietin resistance, and endothelial dysfunction bring about these unfavorable nonatherosclerotic changes to the cardiac myocardium. Uremic cardiomyopathy can further lead to arrhythmogenic complications that result in a high prevalence of sudden cardiac death, which is responsible for almost 40% of the deaths in the ESKD population [2]. Moreover, interstitial fibrotic changes have been detected in the early stages of CKD prior to the development of left ventricular hypertrophy, indicating that these processes start in early CKD. Cardiac magnetic resonance imaging (CMR) is universally considered the gold standard in assessing left ventricular dimensions due to its high spatial resolution, less interobserver variability, and better border definitions [3]. In addition, interstitial fibrosis and edema can be detected by CMR using T1 and T2 mapping [4,5]. We aimed to conduct a systematic review and meta-analysis to provide qualitative and quantitative CMR information about changes in uremic cardiomyopathy.

This article was previously presented as a meeting abstract at the 2023 American Society of Nephrology Annual Scientific Meeting on November 2, 2023.

## Review

Methods

Data Sources and Search Strategy

The following four electronic databases were searched on 3/8/2023 and 3/13/2023 from the inception of each database via a librarian-assisted database search: Medline (PubMed), Embase, Cochrane Central Register of Controlled Trials, and Web of Science (including the Science Citation Index Expanded, Social Science Citation Index, and Arts and Humanities Citation Index). Google Scholar was searched on 3/13/23 using Publish or Perish software, and the first 100 results were selected. We used keywords and controlled vocabulary related to CMR and CKD. A total of 1,295 citations were retrieved. The librarian manually removed duplicates using EndNote's duplicate identification feature [6], leaving 774 results (Table 1).

**Table 1:** Literature search strategy

Database name	Query	Number of search results
Medline (PubMed)	(Cardiac-MRI [tiab] OR Cardiac-magnetic-resonance [tiab] OR Cardiovascular magnetic-resonance [tiab] OR cardiovascular-MRI [tiab]) AND ("Renal Dialysis"[Mesh] OR "Renal Insufficiency, Chronic"[Mesh] OR dialyses [tiab] OR Dialysis [tiab] OR chronic-kidney [tiab] OR chronic-renal [tiab] OR Haemodialysis [tiab] OR hemodialysis [tiab] OR Haemodialyses [tiab] OR hemodialyses [tiab])	241
Embase	('cardiovascular magnetic resonance'/exp OR ((cardiac OR cardiovascular) NEAR/2 (mri OR 'magnetic resonance')):ab,ti) AND ('hemodialysis'/exp OR 'chronic kidney failure'/exp OR dialyses:ab,ti OR dialysis:ab,ti OR 'chronic kidney':ab,ti OR 'chronic renal':ab,ti OR haemodialysis:ab,ti OR hemodialysis:ab,ti OR haemodialyses:ab,ti OR hemodialyses:ab,ti) AND ('article'/it OR 'article in press'/it OR 'review'/it)	569
Cochrane Central Register of Controlled Trials	ID Search Hits #1 (((Cardiac OR Cardiovascular) NEAR/2 (MRI OR magnetic-resonance))):ti,ab,kw 2292 #2 MeSH descriptor: [Renal Insufficiency, Chronic] explode all trees 8473 #3 MeSH descriptor: [Renal Dialysis] explode all trees 6314 #4 (dialys es OR Dialysis OR chronic-kidney OR chronic-renal OR Haemodialysis OR hemodialysis OR Haemodialyses OR hemodialyses):ti,ab,kw 29659 #5 #2 OR #3 OR #4 30939 #6 #1 AND #5 110	110
Web of Science (including Science Citation Index Expanded, Social Science Citation Index, Arts and Humanities Citation Index)	(Cardiac OR Cardiovascular) NEAR/2 (MRI OR magnetic-resonance) (Topic) and dialyses OR Dialysis OR chronic-kidney OR chronic-renal OR Haemodialysis OR hemodialysis OR Haemodialyses OR hemodialyses (Topic) and Article or Review Article or Early Access (Document Types)	275
Google Scholar	Cardiac|Cardiovascular MRI|“magnetic- resonance” Dialyses|Dialysis|“chronic kidney”|“chronic renal”|Haemodialysis|hemodialysis|Haemodialyses|hemodialyses	100

Study Selection

Two reviewers (D. C. and R. R.) independently reviewed (blinded) abstracts and articles with full texts. The decision to include a study was based on the inclusion and exclusion criteria. The reasons for exclusion were recorded, and disagreements were resolved by further discussion and consulting a third author (R. K.). A web-based collaboration software platform (Covidence, Melbourne, Australia) was used for literature screening [7]. The Preferred Reporting Items for Systematic Reviews and Meta-Analysis (PRISMA) guidelines were used to select the final articles [8]. The study protocol was registered in PROSPERO, an international database of systematic reviews, with registration number CRD42023400693.

The inclusion criteria are as follows: (1) patients ≥18 years old with CKD stage 2 or lower (<89 ml/min/1.73 m2) and ESKD; (2) results must report mean values, standard deviations, or confidence intervals (CIs) of CMR values.

The exclusion criteria were as follows: (1) studies reporting CMR findings in (a) myocardial infarction, (b) hypertrophic cardiomyopathy, (c) aortic stenosis, (d) lipid storage diseases, (e) iron accumulation, (f) amyloidosis, and (g) studies evaluating the effects of spironolactone or other agents on systolic function; (2) studies evaluating CMR post-kidney transplantation; and (3) review articles and case series.

Outcomes Assessed

The CMR parameters assessed were left ventricular ejection fraction (LVEF), left ventricular mass index (LVMi), late gadolinium enhancement (LGE), native T1 values, and native T2 values.

Data Extraction

A standardized spreadsheet was used to extract demographic, clinical, and radiological data. All the authors performed data extraction, and the extracted data were verified by two authors (D. C. and R. R.).

Statistical Analysis

Continuous variables were expressed as the mean ± standard deviation, and categorical variables were expressed as percentages. Pooled mean estimates and corresponding 95% CIs for continuous variables were calculated using the inverse variance random-effects DerSimonian-Laird method [9]. The standardized mean difference (SMD) was calculated when comparing two groups using the random effects model. SMDs of 0.2, 0.5, and 0.8 are considered small, medium, and large, respectively [10]. A proportional meta-analysis was performed for dichotomous variables using the random effects model.

Heterogeneity was measured by I2 statistics, with values <30%, 31% to 60%, 61% to 75%, and >75% suggesting low, moderate, substantial, and considerable heterogeneity. Publication bias was ascertained by visual inspection of the funnel plots and by using the Egger test. [11]. Statistical analysis was performed using Comprehensive Meta-Analysis software, version 4 (Biostat, Englewood, NJ, USA).

Quality Assessment and Risk of Bias

The modified Newcastle-Ottawa scale was used to assess bias since only prospective, retrospective studies and single cohorts of RCTs were included in our study. Studies were scored on study selection (representativeness of the exposed cohort, ascertainment of exposure, ascertainment of outcomes at the start), and outcome (assessment of outcome, follow-up time, adequacy of follow-up) [12]. Two authors (D. C. and R. R.) performed the scoring independently. Studies were evaluated for a maximum of 6 points, with a score of 5 suggesting high quality and a score of <5 suggesting low quality.

Results

Search Strategy Results

**Figure 1:**
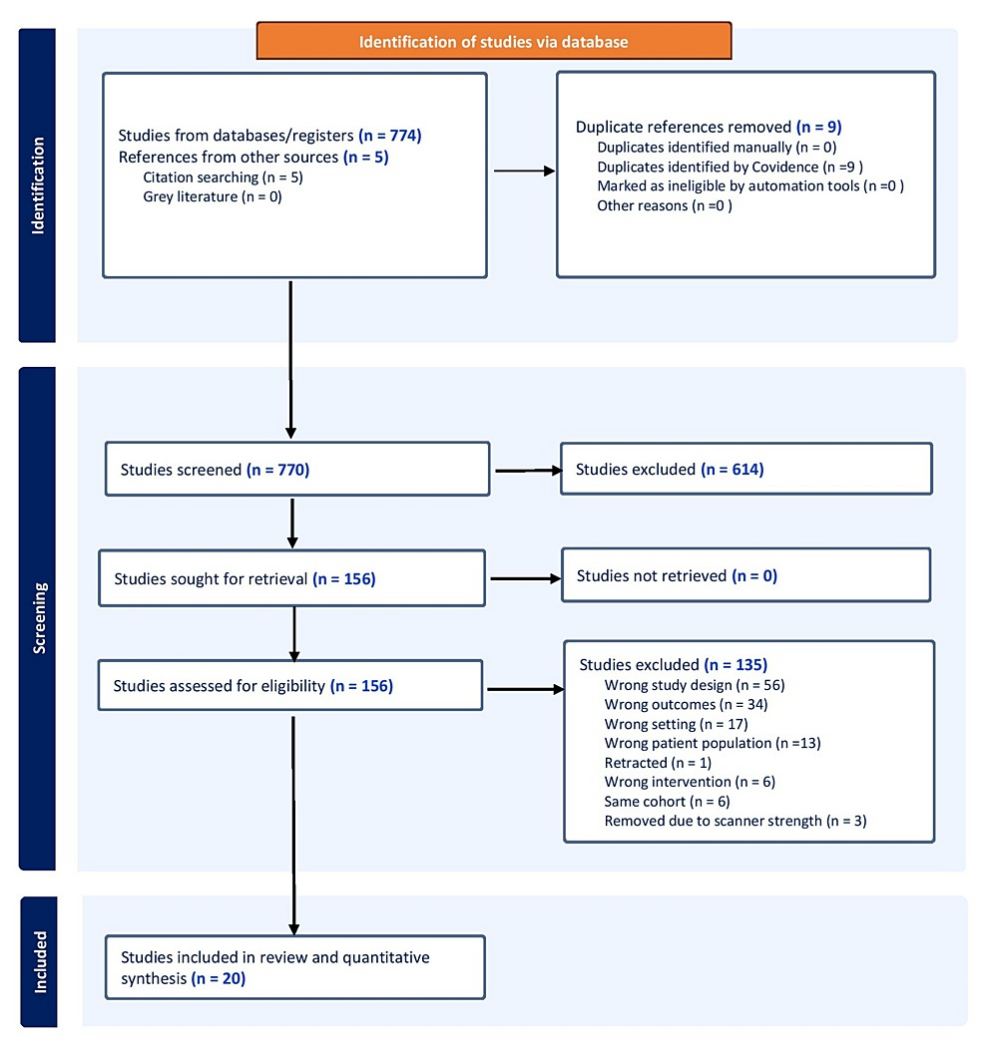
Study selection process according to the PRISMA statement

A total of 779 relevant citations were identified. Following the screening, 156 articles were selected for full-text review. Of these, articles not meeting the inclusion criteria (n=135) and studies with the same cohort (n=6) were excluded. Studies were separated according to the magnetic scanner strength (1.5 or 3 T). We excluded three studies because they utilized both scanner strengths to evaluate their cohort. Finally, 20 studies were selected for the systematic review and meta-analysis. The literature search strategy is presented in Figure 1.

Study Characteristics

The total number of included patients was 1300. The total number of CKD patients was 642, and the number of ESKD patients was 658.

The mean pooled age of CKD patients was 56.87 years (95% CI: 53.28-60.46; I2: 89.21). Males accounted for 65.26% (419). The pooled mean estimated glomerular filtration rate (eGFR) of CKD patients was 33 mL/min/1.73 m2 (95% CI 26.59-39.41; I2 97.09). The pooled mean systolic blood pressure (BP) of CKD patients was 135.35 mmHg (95% CI 130.75-139.96; I2 88.5), and the pooled mean diastolic BP was 80.07 mmHg (95% CI 74.44-85.7; I2 93.4). The number of CKD patients with reported type 2 diabetes was 39.59% (276), hypertension was 51.86% (333), congestive heart failure was 17.79% (124), and coronary artery disease was 14.95% (96).

The mean pooled age of ESKD patients was 55.6 years. Males accounted for 63.3% (417). ESKD patients were on dialysis for a mean of 3.47 years (95% CI 2.91-4.02; I2 98.93%). The mean systolic BP was 142.11 mmHg (95% CI 137.14-147.08; I2 80.73%), and the mean diastolic BP was 79.68 mmHg (95% CI 76.54-82.83; I2 82.39%). The number of patients with reported type 2 diabetes was 30.85% (203), hypertension was 77.81% (512), congestive heart failure was 5.31% (35), and CAD was 11.24% (74). The characteristics, demographic information, CMR measurements, and outcomes of the included studies are detailed in Table 2.

**Table 2:** Patient characteristics and outcomes of 1.5 T and 3 T CMR studies included in the systematic review and meta-analysis Values are expressed as the mean ± standard deviation or as the number (percentage) of subjects BNP: B-type natriuretic peptide; CAD: coronary artery disease; CKD: chronic kidney disease; CMR: cardiac magnetic resonance imaging; EF: ejection fraction; eGFR: estimated glomerular filtration rate; ESKD: end-stage kidney disease; HD: hemodialysis; GLS: global longitudinal strain; HFpEF: heart failure with preserved ejection fraction; LV: left ventricle; LVEF: left ventricular ejection fraction; LVMi: left ventricular mass index; NT-pro-BNP: N-terminal prohormone of brain natriuretic peptide; T1: longitudinal relaxation time; T2: transverse relaxation time; MACE: major adverse cardiac events

Study	Sample size, n	Control, n	Age (years)	Males, n (%)	Systolic BP (mmHg)	CKD stage/dialysis modality	LVEF (%)	Outcomes
Edwards et al. [3]	43	43	57±10	24 (55.8)	127±12	CKD 2-4	71±6	CKD had increased native T1 time. However, no differences in LV mass or EF were seen. Myocardial fibrosis is increased in early CKD and associated with increased GLS.
Chan et al. [13]	120	-	52±14.1	73 (60.8)	-	Hemodialysis	56.3±20.5	More frequent in-center hemodialysis resulted in reduced left ventricular, right ventricular end-systolic, and end-diastolic volume and mass. No changes were observed with ventricular remodeling.
Graham-Brown et al. [14]	124	137	56.6±15.7	91 (73.3)	142.9±21.7	Hemodialysis	53.4±10.2	Increased Native T1 and T2 signal present in dialysis patients compared to controls. Elevated Native T1 may be driven by water and not myocardial fibrosis.
Wang et al. [15]	140	24	64.9±11.9	88 (62.8)	128.3±16.8	CKD 3b-4	62.3±9.7	Individuals with CKD stages 3b-4 have evidence of CMR abnormalities. Albuminuria was not associated with a change in any CMR parameter.
Arcari et al. [16]	276	242	58±21	189 (68.4)	137±21	CKD 3-5	53±17	Adverse cardiac remodeling in CKD could be due to a non-ischemic tissue process driven by fibrosis and myocardial edema. Native T1 with NT-pro-BNP was increased in CKD.
Han et al. [17]	43	28	59±11	28 (65.1)	155±22	Hemodialysis	60±1.2	Hemodialysis patients with preserved EF had increased native T1 and T2 values and decreased GLS compared to controls. Increased T2 values are associated with increased BNP.
Jia et al. [18]	20	32	49±8	12 (60)	143±19	CKD and ESKD on hemodialysis	54±10	Myocardial strain, native T1, and T2 values worsened with the advancing CKD stage.
Kotecha et al. [19]	25	-	63.9±16.3	17 (68)	-	Hemodialysis	56±14.4	Approximately 3-hour sessions of HD are associated with a reduction in LV mass, attributable to a decrease in edema. T1 and T2 values decreased post-dialysis.
Lin et al. [20]	23	42	45±12	12 (52.1)	135.7±17.1	Peritoneal dialysis	59.7±6.5	Increased myocardial native T1 and T2 values and decreased LV global strain were found in ESKD patients with preserved LVEF compared with the healthy controls. In particular, increased myocardial T1 and T2 values were found in ESKD patients who did not show evidence of systolic or diastolic dysfunction on echocardiography.
Ong et al. [21]	30	-	51.6±14.2	16 (53.3)	133±16.8	Hemodialysis	61.3±5.2	Longer dialysis duration of 7-8 hrs/session three times a week improved global circumferential strain in one year.
Parnham et al. [22]	12	10	54±14	5 (41.6)	-	CKD 5	68±13	Myocardial oxygenation was decreased in dialysis patients indicating multivessel microvascular CAD. Myocardial oxygen impairment increased with eGFR.
Qin et al. [23]	52	52	52.6±14.1	33 (63.4)	-	Hemodialysis	56.5±12.6	Native T1 mapping values are significantly higher in ESRD patients, during follow-up (median of 38 months). The global native T1 mapping values predict MACE in hemodialysis patients.
Rutherford et al. [24]	33	28	60.5±16.6	19 (57.5)	142.1±18.1	Hemodialysis	63.2±9.3	Global, septal, and mid-septal T1 times were significantly higher in hemodialysis patients. T1 times and GLS correlated with LV mass.
Stromp et al. [25]	29	33	53.7±12.8	14 (48.2)	-	Hemodialysis	62.8±7.4	Cardiac LVMi and GLS showed an association with matric remodeling, cardiac hypertrophy, and attenuated contractile function.
Zhang et al. [26]	33	52	56.9±15.7	23 (69.6)	132.6±30.9	CKD 3-5	48.1±25	LV strain positively correlated with increasing eGFR.
Hayer et al. [27]	37	-	59±13	20 (54)	132±15	CKD 2-5	70±7	T1 time assessing myocardial fibrosis increased with advancing CKD independent of effects of left ventricular afterload. T1 mapping allows better risk stratification of myocardial disease.
McQuarrie et al. [28]	49	-	56.3±13.8	36 (73.4)	145.7±21	CKD 2-4	69.9±8.9	Proteinuria was independently associated with LVMI in CKD patients.
Odudu et al. [29]	54	29	57±15	39 (72.2)	143±27	Hemodialysis	51±10	CMR within 6 months of starting hemodialysis showed a high prevalence of reduced systolic and diastolic strain, ventricular dyssynchrony, anteroseptal segmental dysfunction, and reduced aortic distensibility even when EF was preserved.
Thompson et al. [30]	15	15	53.9±25.4	10 (66.6)	122.6±33.1	Hemodialysis	-	Long-term hemodialysis patients have more favorable cardiac and vascular structure and function and a trend toward lower native T1 values compared to those who had recently initiated hemodialysis treatment. Long-term dialysis patients also had increased native T1 values as compared to healthy controls but with normal left ventricular mass and volumes. Incident hemodialysis patients had increased native T1 values as well as increased left ventricular mass and reduced function compared to healthy controls.
Zhang et al. [31]	43	-	45.7±15.4	24 (55.8)	137.9±20.8	Hemodialysis and peritoneal dialysis	67.1±6.7	Texture features derived from native T1 mapping had added diagnostic values for HFpEF in ESRD patients.

There were two RCTs and one post hoc study of an RCT, the CKD Optimal Management with Binders and Nicotinamide (COMBINE) trial [13-15]. There were 11 prospective studies, five cross-sectional studies, and one retrospective study [3,16-31]. The included studies are shown in Table 3.

**Table 3:** Summary of included studies and the CMR parameters measured GLS: global longitudinal strain; LA: Left atrium/atrial; LV: Left ventricle; RV: right ventricle; T1: longitudinal relaxation time; T2: transverse relaxation time; PWV: pulse wave velocity; MOLLI: modified Look-Locker inversion recovery; hs-cTnT: high-sensitive cardiac troponin T; NT-pro-BNP: N-terminal prohormone of brain natriuretic peptide; CMR: cardiac magnetic resonance imaging

Study	Year	Country	Study design	Vendor/maker of MRI	Magnetic field strength	CMR parameters reported
Edwards et al. [3]	2015	United Kingdom	Cross-sectional, observational, single center	Siemens Avanto, Germany	1.5 T	LV volume and mass, LA volume, T1 mapping using MOLLI, native T1, LGE
Chan et al. [13]	2013	North America	The frequent hemodialysis network trial cohort, randomized, multicenter	Siemens, Argus, Germany	1.5 T	LV and RV volume and mass
Graham-Brown et al. [14]	2021	United Kingdom	RCT, multicenter, open-label, blinded endpoint	Siemens, Skyra, Germany	3 T	T1 mapping using MOLLI, native T1, native T2 values
Wang et al. [15]	2022	USA	Post hoc study of RCT CKD optimal management with binders and nicotinamide (COMBINE) trial	Siemens, Germany	3 T	LV volume and mass
Arcari et al. [16]	2021	Germany	Prospective, longitudinal, observational, multicenter	Siemens, Skyra, VE11, Germany	3 T	LV volume and mass, LA volume, T1 mapping using Frankfurt Main (FFM)-MOLLI, T2 mapping, hs-cTnT, and NT-pro-BNP
Han et al. [17]	2020	China	Prospective observational, single center	Siemens, Magnetom, Germany	1.5 T	LV volume and mass using, GLS, T1 using MOLLI, and T2 values
Jia et al. [18]	2022	China	Prospective longitudinal observational, single center	Siemens, Germany	1.5 T	LV volume, native T1 mapping using MOLLI
Kotecha et al. [19]	2019	United Kingdom	Prospective, observational, single center	Siemens, Magnetom Aera, Germany	1.5 T	LV volume and mass, LA volume, T1 mapping using MOLLI and T2 mapping
Lin et al. [20]	2021	China	Prospective, observational, single center	Siemens, Magnetom Verio, Germany	3 T	LV volume and mass, native T1 mapping using MOLLI, T2 mapping, cardiac strain
Ong et al. [21]	2018	Canada	Prospective, observational, multicenter	-	1.5 T	LV volume and mass, GLS
Parnham et al. [22]	2015	Australia	Prospective, observational, single center	Siemens, Trio, Germany	3 T	LV volume and mass
Qin et al. [23]	2022	China	Prospective, observational, single center	Philips, Ingenia, Netherlands	3 T	LV volume and mass, myocardial strain values, T1 mapping
Rutherford et al. [24]	2016	United Kingdom	Prospective, observational, multicenter	Siemens, Magnetom Verio, Germany	3 T	LV volume and mass, GLS, T1 mapping using MOLLI
Stromp et al. [25]	2018	USA	Prospective, observational, single center	Siemens, Aera, Germany	1.5 T	LV volume and mass, GLS
Zhang et al. [26]	2021	China	Prospective, observational, single center	Siemens, Skyra, Germany	3 T	LV volume and mass
Hayer et al. [27]	2020	United Kingdom	Cross-sectional, observational, single center	Siemens Avanto, Germany	1.5 T	LV volume and mass, LA volume, T1 mapping using MOLLI, T2 mapping, and inversion recovery imaging after gadolinium
McQuarrie et al. [28]	2011	United Kingdom	Cross-sectional, observational, single center	Siemens Avanto, Germany	1.5 T	LV volume and mass
Odudu et al. [29]	2015	United Kingdom	Cross-sectional, observational, multicenter	GE, Signa HDxt, USA	1.5 T	LV volume and mass, aortic arch pulse wave velocity (PWV)
Thompson et al. [30]	2019	Canada	Cross-sectional, observational, single center	Siemens, Prisma or Skyra, Germany	3 T	LV volume and mass, T1 mapping using MOLLI, T2 mapping

The magnetic strength of CMR was 1.5 T and 3 T in the included studies. Four studies assessed CKD patients using 1.5 T CMR [18,27,28], and seven studies evaluated ESKD patients using 1.5 T CMR [13,17-19,21,25,29]. Four studies utilized 3 T CMR to evaluate CKD patients, and seven studies used 3 T CMR to evaluate ESKD patients [14-16,20,22-24,26,30,31]. Studies with both CKD and ESKD patients in their cohort were done by Jia et al. [18] and Parnham et al. [22]. Thompson et al. divided their ESRD cohorts into those on long-term dialysis and an incident group that had patients start hemodialysis within six months [30].

Quality Assessment

The results of the quality assessment of the 20 studies are described in Table 4.

**Table 4:** Quality assessment of the studies by the Newcastle-Ottawa quality assessment form CMR: cardiac magnetic resonance imaging

Study	Selection				Comparability	Outcome			Score	Quality
	Representativeness of the average adult in the community	Cohort size	Information on CMR outcomes	Cardiac outcomes not present at the start	Any additional intervention after CMR results	Adequate assessment	Follow-up time	Adequacy of follow-up	Max=7, high>5, medium 3-5, low <3	
	Population based: 1; multicenter: 0.5; single-center: 0	>40 patients: 1; 39 to 20: 0.5; <20: 0	Information with clarity: 1; information derived: 0.5	Not present: 1; present: 0	Yes: 1; no: 0	Yes: 1; no: 0	Yes: 1; not mentioned: 0	All patients followed up: 1; >50% followed up: 0.5; <50% followed up OR not mentioned: 0		
Edwards et al. [3]	0	1	1	1	0	1	1	1	6	High
Chan et al. [13]	0.5	1	0.5	1	0	1	1	1	6	High
Graham-Brown et al. [14]	0.5	1	1	1	0	1	1	1	6.5	High
Wang et al. [15]	0.5	1	1	1	0	1	1	1	6	High
Arcari et al. [16]	0.5	1	1	1	0	1	1	1	6.5	High
Han et al. [17]	0	1	1	1	0	1	1	1	6	High
Jia et al. [18]	0	1	1	1	0	1	1	1	6	High
Kotecha et al. [19]	0	0.5	1	1	0	1	1	1	5.5	High
Lin et al. [20]	0	0.5	1	1	0	1	1	1	5.5	High
Ong et al. [21]	0.5	0.5	1	1	0	1	1	1	6	High
Parnham et al. [22]	0	1	1	1	0	1	1	1	6	High
Qin et al. [23]	0	1	1	1	0	1	1	1	6	High
Rutherford et al. [24]	0.5	0.5	0.5	1	0	1	1	1	5.5	High
Stromp et al. [25]	0	0.5	1	1	0	1	1	1	5.5	High
Zhang et al. [26]	0	1	1	1	0	1	1	1	6	High
Hayer et al. [27]	0	0.5	0.5	1	0	1	1	1	5	High
McQuarrie et al. [28]	0	1	1	1	0	1	1	1	6	High
Odudu et al. [29]	0.5	1	0.5	1	0	1	1	1	6	High
Thompson et al. [30]	0	0	1	1	0	1	1	1	5	High
Zhang et al [31]	0	1	1	1	0	1	1	1	6	High

All of the studies scored above five and were considered to be of high quality.

Systolic Function

**Figure 2:**
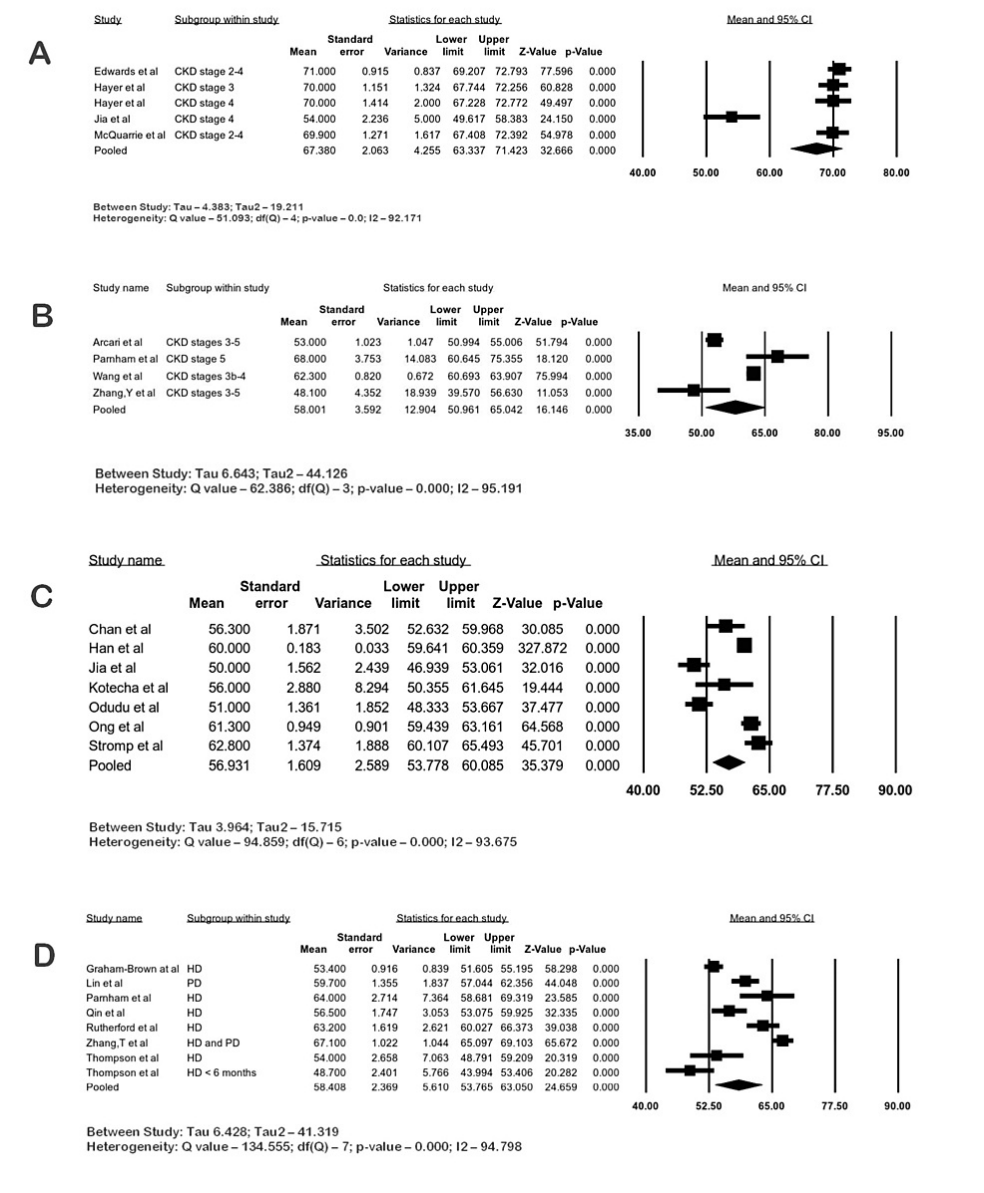
Forest plot of LVEF (%) in CKD by CMR. (A) LVEF in CKD at 1.5 T, (B) LVEF in CKD at 3 T, (C) LVEF in ESKD at 1.5 T, (D) LVEF in ESKD at 3 T CKD: chronic kidney disease; CI: confidence interval; ESKD: end-stage kidney disease; HD: hemodialysis; PD: peritoneal dialysis; LVEF: left ventricular ejection fraction; CMR: cardiac magnetic resonance imaging Edwards et al. [3], Chan et al. [13], Graham-Brown et al. [14], Wang et al. [15], Arcari et al. [16], Han et al. [17], Jia et al. [18], Kotecha et al. [19], Lin et al. [20], Ong et al. [21], Parnham et al. [22], Qin et al. [23], Rutherford et al. [24], Stromp et al. [25], Zhang et al. [26], Hayer et al. [27], McQuarrie at al. [28], Odudu et al. [29], Thompson et al. [30], Zhang et al. [31]

All studies reported LVEF. The mean LVEF of CKD patients measured by 1.5 T CMR was 67.38% (95% CI 63.33-71.42; I2 92.17%), and the mean LVEF of CKD patients measured by 3 T CMR was 58% (95% CI 50.96-65.04; I2 95.19%). The mean LVEF of ESKD patients measured by 1.5 T CMR was 56.93% (95% CI 53.77-60.08; I2 93.67%), and the mean LVEF of ESKD patients measured by 3 T CMR was 58.4% (95% CI 53.76-63.05; I2 94.79%). Heterogeneity was considerable in the meta-analysis. The forest plots are shown in Figure 2A-2D.

Left Ventricular Mass Index

A total of 16 studies evaluated the LVMi by cine imaging using a balanced steady-state free precession (SSFP) sequence [3,14,16,17,19,20-25,27-31].

**Figure 3:**
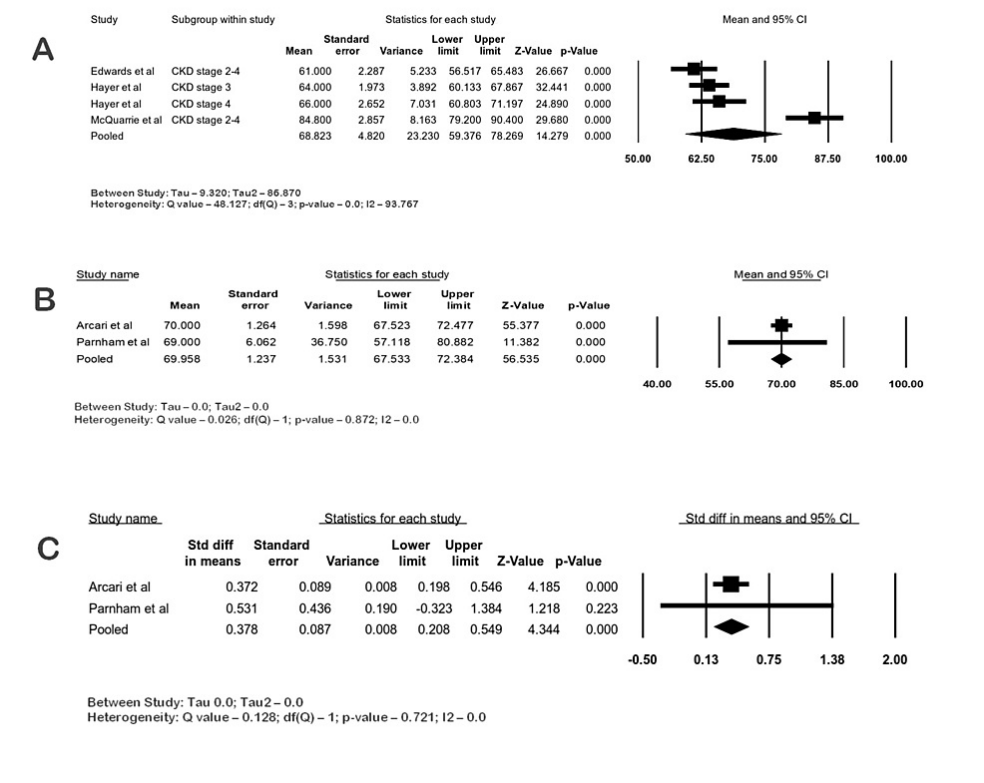
Forest plots of LVMi (g/m2) in CKD by CMR. (A) LVMi in CKD at 1.5 T, (B) LVMi in CKD at 3 T, (C) LVMi in CKD vs. control at 3 T CKD: chronic kidney disease; CI: confidence interval, LVMi: left ventricular mass index; CMR: cardiac magnetic resonance imaging Edwards et al. [3], Arcari et al. [16], Parnham et al. [22], Hayer et al. [27], Mcquarrie et al. [28]

A meta-analysis of three studies with 161 patients showed that the mean LVMi of CKD patients at 1.5 T was 68.82 g/m2 (95% CI 59.37-78.26; I2 93.76). In the analysis of two studies with 288 CKD patients, the mean LVMi at 3 T was 69.95 g/m2 (95% CI: 67.53-72.38; I2 0). The SMD of LVMi between the CKD and control groups at 3 T was 0.37 (95% CI: 0.2-0.54; I2 0; p-value <0.05), showing a moderate difference with low heterogeneity between studies. The forest plots are shown in Figure 3A-3C.

**Figure 4:**
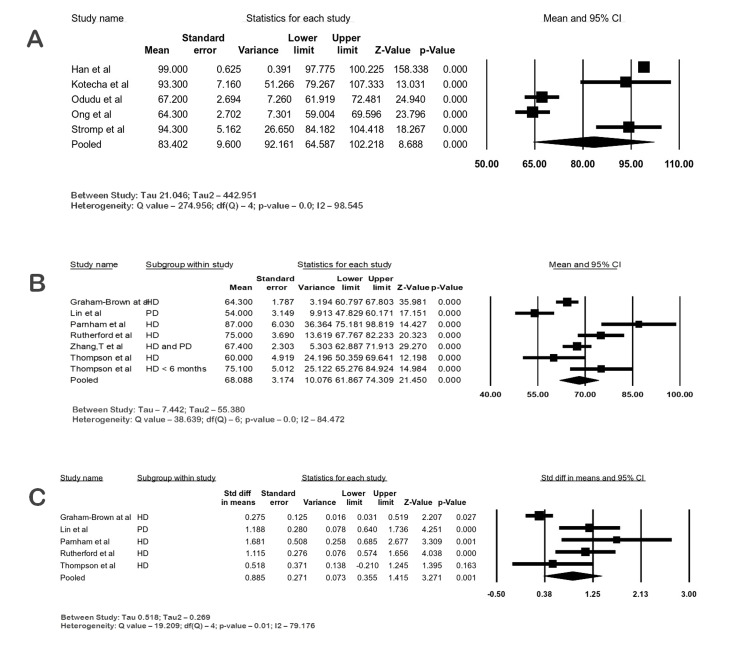
Forest plots of LVMi (g/m2) in ESKD by CMR. (A) LVMi in ESKD at 1.5 T, (B) LVMi in ESKD at 3 T, (C) LVMi in ESKD vs. control at 3 T CI: confidence interval; ESKD: end-stage kidney disease; HD: hemodialysis; PD: peritoneal dialysis, LVMi: left ventricular mass index, CMR: cardiac magnetic resonance imaging Graham-Brown et al. [14], Han et al. [17], Kotecha et al. [19], Lin et al. [20], Ong et al. [21], Parnham et al. [22], Rutherford et al. [24], Stromp et al. [25], Odudu et al. [29], Thompson et al. [30], Zhang et al. [31]

Five studies with 181 ESKD patients were analyzed, and their mean LVMi at 1.5 T CMR was 83.40 g/m2 (95% CI: 64.58-102.21; I2 98.54) and at 3 T CMR was 68.08 g/m2 (95% CI: 61.86-74.30; I2 84.47%). The SMD of LVMi between the ESKD and control groups was 0.88 (95% CI: 0.35-1.41; I2 79.1; p-value 0.001), denoting a large difference. Heterogeneity was considerable in the meta-analysis. The forest plots of LVMi are shown in Figure 4A-4C.

McQuarrie et al. looked at LVMi and proteinuria in people with diabetic nephropathy and IgA nephropathy. They found that proteinuria was linked to a higher left ventricular mass in people with CKD stages 2-4. The mean LVMi in their study was 84.8 g/m2, the mean protein-creatinine ratio was 82.5 (23-236) mg/mmol, and the mean LVEF was 69.9%. LVMi was higher in males and lower in patients on renin angiotensin aldosterone system inhibitors [28]. Rutherford et al. showed that patients on hemodialysis had increased LVMi, a lower peak global longitudinal strain, and increased global native T1 when compared to healthy controls [24].

Late Gadolinium Enhancement

**Figure 5:**
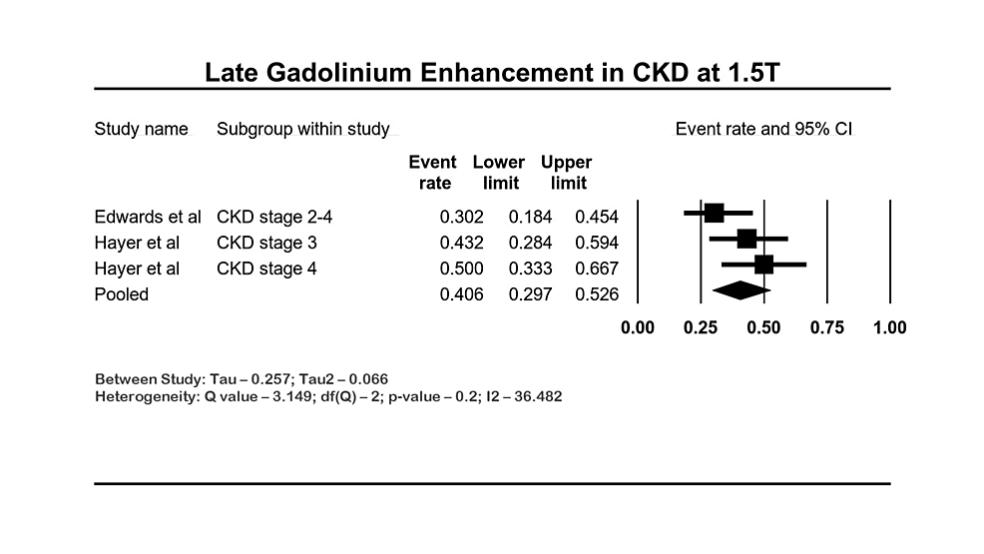
Forest plot of rates of LGE in CKD by CMR CKD: chronic kidney disease; CI: confidence interval, LGE: late gadolinium enhancement, CMR: cardiac magnetic resonance imaging Edwards et al. [3], Hayer et al. [27]

There were four studies with CKD stages between 2 and 4 reporting LGE after gadobutrol contrast (a nonionic group II agent), and analysis was performed on 112 patients from two studies [3,27]. All these studies excluded patients who had contraindications to gadolinium contrast. The proportion of CKD patients with LGE was 40.6% (95% CI: 29.7-52.6; I2: 36.48%). Heterogeneity was moderate in the meta-analysis evaluating LGE. The forest plot of LGE is shown in Figure 5.

T1 Mapping

A total of 11 studies assessed T1 mapping, of which 10 used a modified Look-Locker inversion recovery (MOLLI) to perform native T1 mapping [3,14,16-20,23,24,27,30]. Arcari et al. used a modified in-house variant of Frankfurt Main (FFM)-MOLLI [16]. Three studies measured T1 mapping by 1.5 T CMR in CKD patients [18,27,32]. Three studies studied T1 mapping in ESRD patients using 1.5 T CMR, and four studies used 3 T CMR for T1 mapping in ESRD patients [14,17-19,23,30,33]. The study by Hayer et al. had CKD patients in their cohort, and they were divided into different groups based on eGFR [27].

T1 Values in CKD

**Figure 6:**
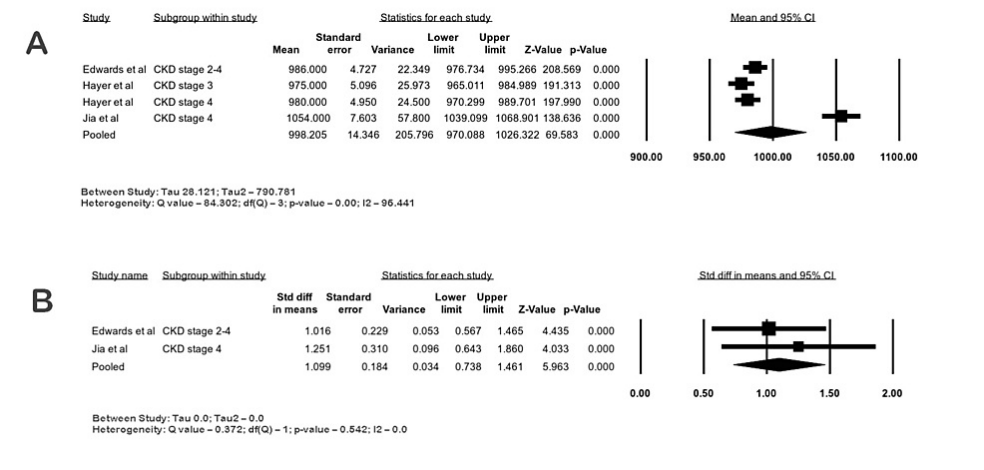
Forest plots of septal T1 (ms) relaxation times in CKD by CMR. (A) Native septal T1 in CKD at 1.5 T, (B) native septal T1 in CKD vs. control at 1.5 T CKD: chronic kidney disease; CI: confidence interval, CMR: cardiac magnetic resonance imaging Edwards et al. [3], Jia et al. [18], Hayer et al. [27]

In three studies with 132 CKD patients, the pooled mean native septal T1 at 1.5 T CMR was 998.2 ms (95% CI 970.08-1026.32; I2 96.44%). A meta-analysis of two studies comparing 63 CKD patients with 75 controls was conducted, and the overall SMD of native septal T1 of CKD and controls at 1.5 T was 1.099 (95% CI: 0.73-1.46; I2 0%; p-value <0.05), suggesting a large difference with small heterogeneity between the two groups. The forest plots are shown in Figure 6A-6B.

T1 Values in ESKD

**Figure 7:**
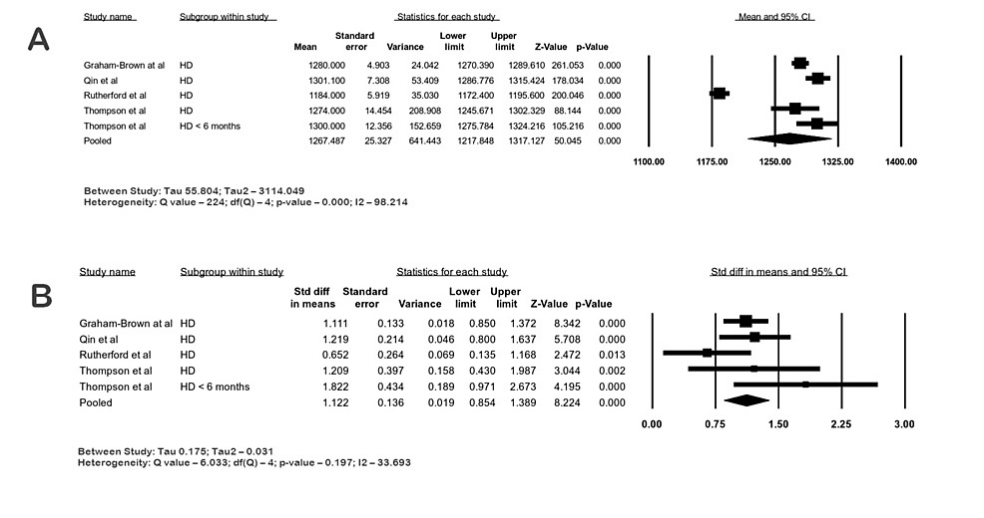
Forest plots of septal T1 (ms) relaxation times in ESKD by CMR. (A) Native septal T1 in ESKD at 3 T, (B) native septal T1 in ESKD vs. control at 3 T ESKD: end-stage kidney disease; CI: confidence interval; HD: hemodialysis; PD: peritoneal dialysis Graham-Brown et al. [14], Qin et al. [23], Rutherford et al. [24], Thompson et al. [30]

The mean native septal T1 of 239 ESKD patients from four studies at 3 T CMR was 1267.48 ms (95% CI: 1217.84-1317.12; I2 98.21%). The SMD of native septal T1 values in four studies accounting for 239 ESKD patients and 232 controls was 1.12 (95% CI: 0.85-1.38; I2 33.69; p-value <0.05), suggesting a large difference with moderate heterogeneity. The forest plots are shown in Figure 7A-7B.

T2 Mapping

A total of three studies evaluated T2 mapping in ESRD patients using 1.5 T, and three studies used 3 T in ESKD patients [14,17-20,30].

T2 Values in ESKD Patients

**Figure 8:**
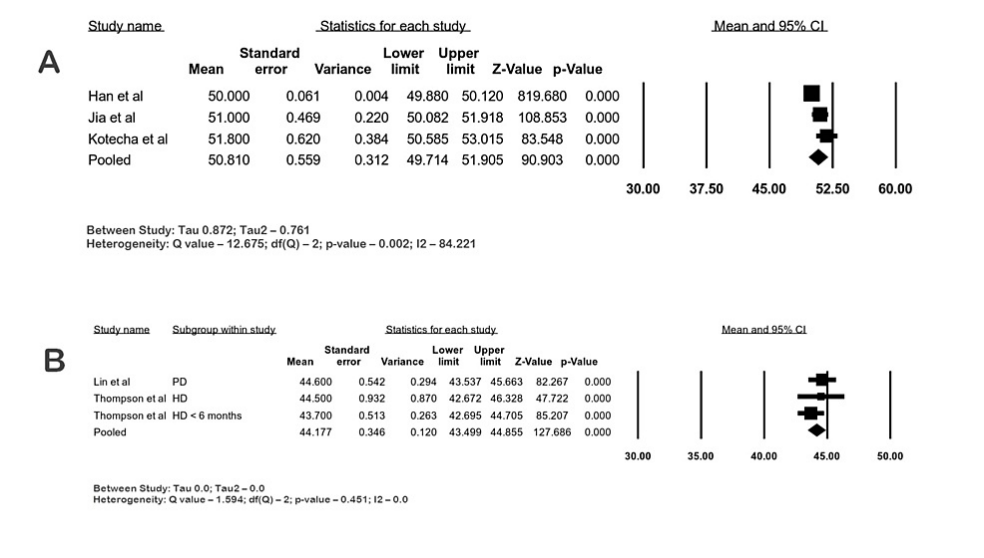
Forest plots of T2 relaxation times (ms) in ESKD by CMR. (A) Global native T2 in ESKD at 1.5 T, (B) global native T2 by bSSFP in ESRD at 3 T bSSFP: balanced steady-state free precession; CI: confidence interval; ESKD: end-stage kidney disease; HD: hemodialysis; PD: peritoneal dialysis Han et al. [17], Jia et al. [18], Kotecha et al. [19], Lin et al. [20], Thompson et al. [30]

Three studies with 109 ESKD patients evaluated the T2 using single-shot SSFP in 1.5 T CMR. The mean T2 value was 50.81 ms (95% CI: 49.71-51.9; I2 84.22%), and the heterogeneity was considerable. Two studies with 53 patients evaluated the T2 using balanced SSFP in 3 T CMR. The mean T2 was 44.17 ms (95% CI: 43.49-44.85; I2 0%) with low heterogeneity. The forest plots for T2 values are shown in Figure 8A-8B.

Publication bias

**Figure 9:**
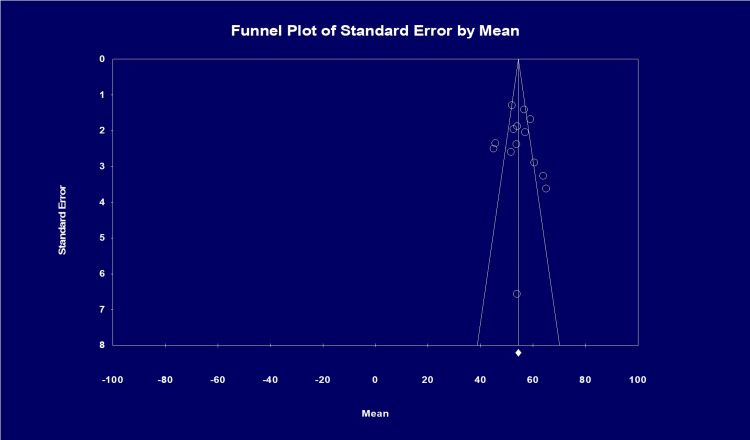
Analysis of publication bias in studies with ESKD patients Egger’s test for a regression intercept gave a 1-tailed p-value of 0.326, indicating no evidence of publication bias. (The intercept (B0) is 0.85422, 95% CI (-3.18464, 4.89308), with t=0.46082, df=12. The 2-tailed p-value is 0.65317)

Based on visual inspection of the funnel plot and by using the Egger regression test, there was no evidence of publication bias in the studies (Egger’s p-value=0.32). The funnel plot is shown in Figure 9.

Discussion

It has been scientifically proven by several studies, and we reinforce that patients with CKD and ESKD have increased LVMi in the presence of a preserved ejection fraction. Furthermore, there were increased T1 values and the presence of LGE, indicating an increased burden of fibrosis in this population.

Two-dimensional (2D) M-mode echocardiography overestimates left ventricular mass and has other limitations, such as interobserver variability and dependence on volume status to determine LV volume. For these reasons, multiparametric CMR analyses are increasingly utilized to assess LV function and diagnose specific cardiomyopathies [34]. The cine technique with balanced SSFP is used to measure the size and mass of the LV. SSFP produces bright signals from still tissues while reducing signals from moving tissues and blood. CMR can accurately measure LVMi, providing valuable data in patients without symptomatic cardiac disease or dilated cardiomyopathy [5].

Up to 70% of ESKD patients have left ventricular hypertrophy. Since increased LVMi has been directly related to increased mortality, many studies have evaluated its relationship to dialysis. Conventional thrice-weekly hemodialysis causes LVMi regression in only approximately 50% of patients [35]. The Frequent Hemodialysis Network compared six times per week to three times per week of in-center hemodialysis. The arm with six times per week of dialysis had a greater reduction in left and right ventricular systolic and diastolic volumes and mass. In the Frequent Hemodialysis Network nocturnal trial, patients with preexisting uremic cardiomyopathy had a 22% decrease in LVMi with nocturnal home HD compared to conventional HD [13].

LGE has been used due to its high sensitivity to detect diffuse myocardial fibrosis and scarring. However, its use may be limited in patients with advanced CKD due to concerns about nephrogenic systemic fibrosis. Areas of fibrosis appear brighter after gadolinium contrast administration due to the increased volume of distribution of the contrast and the prolonged washout. The presence of subendocardial LGE represents prior infarction, and diffuse fibrosis could represent changes due to uremic cardiomyopathy. Many studies in CKD and ESKD patients have shown increased LGE [32,36]. A study by Schietinger et al. demonstrated the presence of LGE in 79% of hemodialysis patients. Most of these LGEs were not related to prior infarcts but were associated with dysfunctional LV segments [37]. In our meta-analysis, LGE was present in more than one-third of the patients.

Native T1 values are capable of spotting early and subtle myocardial changes that are invisible to the naked eye on grayscale images. They can differentiate between normal and infarcted areas of the myocardium. This quantitative technique to detect focal fibrosis is based on the longitudinal recovery time of excited hydrogen atoms in various tissues. T1 mapping is obtained by combining a series of T1 relaxation times. This has been reliably used in the diagnosis of cardiomyopathies, quantification of areas of fibrosis, prognostic determination, and guidance of therapies [38]. Several studies have reported increased T1 values in CKD and ESRD patients [39]. Myocardial fibrosis unrelated to ischemia occurs early in uremic cardiomyopathy, and this increases with the severity of CKD [40]. T1 mapping can be used as a surrogate biomarker to evaluate fibrosis in a non-invasive manner. Furthermore, it has been shown that elevated T1 values and biopsy-measured fibrosis had a good correlation. Qin et al. showed that T1 mapping could predict major adverse cardiac events in hemodialysis patients [23]. Early detection of fibrosis could provide the necessary therapeutic interventions since systolic and diastolic function over the long term culminates in dilated cardiomyopathy and ultimately leads to congestive heart failure. Hayer et al. found that cardiac fibrosis in CKD patients without diabetes was independent of hypertension and aortic distensibility [27]. Edwards et al. found that T1 values were higher in CKD patients than in hypertensive patients and healthy controls [3]. Lin et al. demonstrated that ESKD patients on peritoneal dialysis with normal diastolic function had higher T1 and T2 values, and no difference in values was found between patients with or without hypertension or anemia [20]. In a study by Puntmann et al., increased native T1 values were predictive of all-cause mortality and heart failure in nonischemic cardiomyopathy [41]. Increased iron load, genetic cardiomyopathies, lipid storage diseases, prior infarcts, cardiomyopathies due to aortic stenosis, and amyloidosis can also cause abnormal T1 values, and these studies were excluded from our meta-analysis. Increased T1 values are usually seen with 3 T scanners compared to 1.5 T scanners due to the increased magnetic field strength [42]. Our meta-analysis showed increased T1 values in CKD and ESKD patients compared to controls.

T2 mapping measures the course of radiofrequency-excited hydrogen atoms and their transverse relaxation. T2 mapping has increased accuracy compared with T2-weighted imaging. Increased edema states in the myocardium cause longer T2 relaxation times, so they are indicative of the volume status of patients. They can also detect areas of inflammation and are currently utilized to diagnose myocarditis, drug and chemotherapeutic agent toxicities, and cardiac transplant rejection [5]. Studies by Arcari et al. and Kotecha et al. showed a reduction in T2 values post-dialysis [16,19]. Han et al. demonstrated that T2 values correlated with T1 values in hemodialysis patients [17]. T2 mapping has been validated in myocarditis, myocardial infarction, and other cardiomyopathies [43].

The use of CMR has some limitations. CMR may not be available in all facilities, and it also requires longer examination times. It may not be suitable for claustrophobic patients, patients who have metallic intraocular implants, cochlear implants, neurostimulation systems, or other ferromagnetic objects. Despite these drawbacks, it remains a robust and accurate tool to detect LV remodeling.

Limitations

Our study has some limitations. First, since meta-analysis inherently relies on the studies included, the limitations and biases of individual studies are incorporated into the analysis. Second, most of the studies were observational studies, and some had a small sample size. Third, the timing of dialysis and the volume status varied across studies, although most studies performed CMR post-dialysis. Fourth, the presence of other co-morbidities could also contribute to fibrotic changes in the myocardium. Finally, studies were divided into 1.5 and 3 T scanners, but the use of different CMR scanners, protocols, and vendors in the studies can yield different CMR values.

## Conclusions

Our meta-analysis findings suggest that the use of CMR in CKD and ESKD patients can diagnose early unfavorable LV remodeling, despite the heterogeneity in the included studies being a limitation. Myocardial LVMi and native septal T1 values are increased in CKD and ESKD compared to controls. T1 mapping can detect cardiac fibrosis in the early stages of CKD and ESKD and can help clinicians stratify subclinical disease risk. Further, large, blinded RCTs with longer follow-ups are necessary to study LV changes in detail and their relationship with dialysis. There is a need to standardize threshold values while using T1 mapping with different scanner strengths. Further studies are also needed to validate T1 and T2 mapping techniques in CKD and ESKD populations.
